# Antenatal magnesium individual participant data international collaboration: assessing the benefits for babies using the best level of evidence (AMICABLE)

**DOI:** 10.1186/2046-4053-1-21

**Published:** 2012-03-21

**Affiliations:** 1 Australian Research Centre for Health of Women and Babies (ARCH), Discipline of Obstetrics and Gynaecology, Women's and Children's Hospital, The University of Adelaide, Adelaide, Australia

**Keywords:** Preterm birth, Magnesium sulphate, Individual patient data, Cerebral palsy, Meta-analysis

## Abstract

**Background:**

The primary aim of this study is to assess, using individual participant data (IPD) meta-analysis, the effects of administration of antenatal magnesium sulphate given to women at risk of preterm birth on important clinical outcomes for their child such as death and neurosensory disability. The secondary aim is to determine whether treatment effects differ depending on important pre-specified participant and treatment characteristics, such as reasons at risk of preterm birth, gestational age, or type, dose and mode of administration of magnesium sulphate.

**Methods:**

**Design**

The Antenatal Magnesium Individual Participant Data (IPD) International Collaboration: assessing the benefits for babies using the best level of evidence (AMICABLE) Group will perform an IPD meta-analysis to answer these important clinical questions.

**Setting/Timeline**

The AMICABLE Group was formed in 2009 with data collection commencing late 2010.

**Inclusion Criteria**

Five trials involving a total 6,145 babies are eligible for inclusion in the IPD meta-analysis.

**Primary study outcomes**

For the infants/children: Death or cerebral palsy. For the women: Any severe maternal outcome potentially related to treatment (death, respiratory arrest or cardiac arrest).

**Discussion:**

Results are expected to be publicly available in 2012.

## Background

### Preterm birth and neurological outcome: The burden of disease

Babies born preterm compared with those born at term have a higher chance of dying in the first few weeks of life. Babies who survive have a greater risk of neurologic impairment, such as cerebral palsy, blindness, deafness, or cognitive dysfunction (either intellectual impairment or developmental delay), and a greater risk of substantial disability as a result of these neurologic impairments [1,2]. Moreover, as the rate of preterm birth in many countries has been rising, now up to 13% in the United States [3] and 8% in Australia [4], more babies are at risk of death and adverse neurological outcomes. Cerebral palsy and cognitive dysfunction are the most frequent neurologic impairments, and any therapy that can reduce their prevalence would have a substantial effect on reducing overall neurologic impairments and disabilities in surviving preterm infants.

Cerebral palsy is a term which includes a number of different diseases or conditions that can arise at any time during brain development. Cerebral palsy involves a disorder of movement or posture, or both, and a disorder of motor function which is permanent but may change over time [5]. The cerebral palsies remain the most frequent cause of severe motor disability in childhood with a background prevalence of two per thousand live births [5]. Over 90% of affected children with cerebral palsy are expected to survive to 20 years of age [6], contributing substantially to the burden of illness into adulthood.

Very preterm birth (less than 32 weeks' gestation) and very low birthweight (less than 1500 g) are principal risk factors for cerebral palsy [7,8], responsible for between 17% to 32% of all cases of cerebral palsy.

Evidence from population-based registries generally shows increasing rates of cerebral palsy as gestational age decreases; with overall rates of cerebral palsy now generally considered to be stable over time [9-11] or decreasing [12]. Intraventricular haemorrhage (IVH) is a known risk factor for the later development of cerebral palsy [13,14] with the rates of IVH and periventricular leukomalacia, another risk for cerebral palsy, increasing the earlier the gestational age at birth.

### Biological plausibility and early evidence for effect of antenatal use of magnesium sulphate on neurodevelopment

In humans, magnesium is essential for key cellular processes, including glycolysis, oxidative phosphorylation, protein synthesis, DNA and RNA aggregation and maintenance of plasma membrane integrity [15-17]. Magnesium favourably affects mechanisms implicated in cell death by decreasing proinflammatory cytokines or free radicals produced during hypoxic-ischaemic reperfusion and inflammatory diseases of pregnancy [15,18]. Magnesium prevents excitotoxic calcium-induced injury [19], by a non-competitive voltage-dependent inhibition of the N-methyl-D-aspartate receptor to glutamate which reduces calcium entry into the cell [20]. Magnesium has some beneficial haemodynamic effects including stabilising blood pressure during the first two days of life in preterm neonates [21], and may increase cerebral blood flow by reducing constriction of the cerebral arteries [22].

Transplacental transfer of magnesium is rapid with magnesium concentrations increased in fetal serum within one hour of maternal intravenous administration [23].

The first report that antenatal magnesium sulphate was associated with a reduced risk of IVH, from 18.9% to 4.4%, in very low birth weight babies (less than 1500 g) came in 1992 [13] followed by a case-control analysis from the California Cerebral Palsy project investigating whether in utero exposure to magnesium sulphate was associated with a lower prevalence of cerebral palsy in low birth weight infants [24]. Cases were children with cerebral palsy who were singletons and whose birthweight had been less than 1500 g. Controls were randomly sampled from live births of less than 1500 g from the same birth populations. Magnesium sulphate given to the mother during labour was associated with a dramatic reduction in the risk of cerebral palsy (odds ratio 0.14; 95% confidence interval (CI) 0.05 to 0.51).

Other observational studies have supported a reduction in cerebral palsy in preterm babies after maternal administration of magnesium sulphate [25-27] and some have found a reduction in the risk of IVH [25,28] and perinatal mortality [29]. However, not all observational studies have reported benefit on the risk of IVH for antenatal magnesium sulphate [30-32], cerebral palsy [31,33,34], or perinatal mortality [30].

Magnesium sulphate, by its peripheral vasodilator effects when infused intravenously, produces a sensation of warmth, flushing and sweating. Reported maternal side-effects, related to dosage and speed of infusion, include nausea, vomiting, headache and palpitations. Magnesium sulphate also acts as a neuromuscular blocking agent that causes abolition of tendon reflexes [35]. Magnesium could aggravate the cardiovascular or neuromuscular side-effects of other drugs such as betamimetics, calcium-channel blockers, digitalis and gentamicin. Infusion to concentrations above the recommended therapeutic range can lead to respiratory depression, respiratory arrest, cardiac arrest and death. For the neonate, hypermagnesaemia can lead to hyporeflexia, poor sucking, and, rarely, respiratory depression needing mechanical ventilation [36,37].

### Summary of the Cochrane systematic review (aggregate data)

The Cochrane systematic review assessing the use of magnesium sulphate for women at risk of preterm birth for neuroprotection of the fetus [38] includes five trials (6145 babies): two from the US; the MagNet Trial [39] and the Beam Trial [40], one from Australia and New Zealand; the ActoMgSo4 Trial [41], one from France; the PreMag Trial [42], and one that was worldwide, but predominantly from developing countries; the Magpie Trial [43]. The first four trials specifically targeted women likely to give birth early and magnesium was used for neuroprotection, although one study, the MagNet trial [39], also had a tocolytic arm. The fifth study, the Magpie Trial was designed to prevent eclampsia in women with pre-eclampsia and included women at all gestational ages. Data from the Magpie Trial study relevant to women randomised before birth and at less than 37 weeks were provided by the authors for inclusion in the Cochrane review.

There was diversity in the inclusion and exclusion criteria for the five included trials. In particular there was a wide variation in reasons women were at risk of preterm birth (preterm labour, preterm prelabour rupture of the membranes, pre-eclampsia), the gestational age women were eligible, time of treatment prior to expected preterm birth and drug regimens (Table 1).

**Table 1:** Eligible trials with their inclusion criteria and magnesium regimens as at September 2011

Trial	GA at entry (weeks)	Treatment intent	Magnesium sulphate regimen	Comparator
**Mittendorf 2002 **[39]	25 to 33	Tocolysis (cervical dilatation < 4 cm) or;	4 g bolus followed by 2 to 3 g/hour maintenance	Alternative tocolytic
		
		Neuroprotection of the fetus/infant/child (dilatation ≥ 4 cm)	4 g bolus (no repeats)	Placebo

**Rouse 2008 **[40]	24 to 32	Neuroprotection of the fetus/infant/child	6 g over 20-30 mins followed by 2 g/hr (retreatment permitted)	Placebo

**Crowther 2003 **[41]	less than 30	Neuroprotection of the fetus/infant/child	4 g over 20 mins, then 1 g/hour for up to 24 hours or birth (no repeats)	Placebo

**Marret 2008 **[42]	less than 33	Neuroprotection of the fetus/infant/child	4 g (no repeats)	Placebo

**The Magpie Trial Collaborative Group 2002 **[43]	less than 37	Neuroprotection of the pre-eclamptic mother	4 g over 10-15 mins, then either 1 g/hour for 24 hours, or 5 g every 4 hours IM for 24 hours (no repeats)	Placebo

Paediatric mortality was reported in five trials with 6145 babies. Antenatal magnesium sulphate treatment had no overall significant effect on total paediatric mortality overall (risk ratio (RR) 1.04, 95% CI 0.82 to 1.17, five trials, 6145 infants). While results between treatment intent did not differ, there was substantial heterogeneity (I^2 ^= 45%) between studies for this outcome, largely due to the other intent (tocolytic) arm of the MagNet study (RR 15.8, 95% CI 0.93 to 267, 106 infants).

Cerebral palsy was reported in five trials with 6145 infants. There was a 32% relative reduction in risk of cerebral palsy with antenatal magnesium sulphate treatment, (overall RR 0.68, 95% CI 0.54 to 0.87). This remained significant within the neuroprotective intent group (RR 0.71, 95% CI 0.55 to 0.91, four trials, 4446 infants). Overall, the number needed to treat to prevent one case of cerebral palsy was 63 (95% CI 39 to 172).

Antenatal magnesium sulphate treatment had no overall significant effect on the combined outcome of paediatric mortality or cerebral palsy, but there was substantial heterogeneity overall (I^2 ^= 51.3%). However, there was a significant reduction in the combined outcome of paediatric mortality or cerebral palsy in the neuroprotection subgroup (RR 0.85, 95% CI 0.74 to 0.98, four trials, 4446 infants), with little evidence of heterogeneity (I^2 ^= 5.3%). The number needed to treat for an extra survivor free of cerebral palsy in the neuroprotective subgroup was 41 (95% CI 22 to 357).

### Need for an individual participant data meta-analysis

Aggregate data analysis methods such as those used in the Cochrane review are limited because, for example, results for women with different levels of risk have been averaged, potentially concealing any different effects by reasons that women were considered to be at risk for preterm birth. Similarly wide within-trial variation in different degrees of effect of magnesium sulphate by gestational age is concealed by aggregation. Aggregated outcomes such as 'the number of children developing cerebral palsy' include a range of severity of the disorder. The published aggregate data is variable in completeness of reporting outcomes and in the definitions used within the trials. Not all trials have published the same combined outcomes and very few subgroup analyses using similar subgroup categories (such as gestational age) have been published, making aggregation of these results impossible.

Many questions remain. Is antenatal magnesium sulphate treatment more effective in some women by reason of their risk of preterm birth? What is the gestational age range for maximal benefit? What dose and timing prior to birth of magnesium is best? Is maintenance treatment or re-treatment necessary?

The best way to address the limitations of the aggregate meta analysis is to use the large amount of existing trial data and conduct an individual participant data (IPD) review and meta-analysis, rather than undertake another trial which would need to be much larger than any of the existing trials to be able to address the multiple subgroup analyses with sufficient power.

Analysis of thoroughly checked and updated data from individual participants in all the available randomised trials has been described as the gold standard in systematic reviews [44]. Estimates of treatment effects are often different from those obtained from aggregate published data due to the ability to include additional unpublished data and to categorise all participants according to standardised definitions. The methods and advantages of IPD review have been well described [45,46].

Advantages that address the limitations of aggregate data analyses reported include the ability to:

• undertake subgroup analyses to examine important hypotheses about differences in treatment effect that cannot be achieved with the published aggregate data,

• explore interactions between treatment and participant-level characteristics,

• collect uniformly defined, relevant data for all trials.

An integral component of conducting this IPD meta-analysis is the formation of an international collaborative group of trialists where all researchers endorse the IPD protocol and provide data from their trials. This generates additional benefits that include:

• more complete identification of trials and of trial details,

• compliance with standard definitions, provision of missing data on characteristics of trials, all women who were randomised and their babies, and outcomes,

• more balanced interpretation, endorsement and global dissemination of results, and

• better clarification and consensus on future research needed with the opportunity for ongoing international collaborations.

## Objectives

To assess, using IPD meta-analyses, the effects of administration of antenatal magnesium sulphate given to women at risk of preterm birth on important clinical outcomes, and whether treatment effects differ depending on important prespecified participant and treatment characteristics:

• the reason the woman was considered to be at risk of preterm birth (such as preterm labour, hypertensive disease of pregnancy, antepartum haemorrhage, presence or absence of ruptured membranes at trial entry),

• the primary reason antenatal magnesium sulphate treatment was given (such as neuroprotection of the fetus, pre-eclampsia, or tocolysis),

• the number of babies in utero (singleton or multiple),

• the gestational age when antenatal magnesium sulphate treatment was given,

• the time prior to birth antenatal magnesium sulphate treatment was given,

• the type, mode of administration and dosage of antenatal magnesium sulphate planned and given,

• whether maintenance treatment with antenatal magnesium sulphate was used, and

• whether repeat antenatal magnesium sulphate treatment was used.

## Methods/design

### Study design

IPD meta-analysis.

### Criteria for the studies for inclusion and exclusion

Studies, published or unpublished, will be considered eligible if they randomised women considered at risk of preterm birth (less than 37 weeks' gestation) to either magnesium sulphate or a suitable control treatment with adequate allocation concealment. Trials will be included if the primary aim of the study was to prevent neurologic abnormalities in the unborn baby, or if the primary aim was otherwise but long-term neurological outcomes were reported for the infants. Quasi-random study designs will be excluded. See Table 1 for a summary of the eligible trials as at September 2011.

Eligibility of trials will be assessed independently and unblinded for author and journal by two members of the AMICABLE IPD Project Team. Any differences in opinion regarding eligibility will be resolved by discussion. If IPD are unavailable from any eligible trial, the trial will be included in the review and aggregated and stratified data will be used for sensitivity analyses were possible.

### Search strategy to identify potential trials

We will use the search strategy developed by the Cochrane Pregnancy and Childbirth Review Group for the Cochrane review, which identifies trials from:

1. quarterly searches of the Cochrane Central Register of Controlled Trials (CENTRAL);

2. weekly searches of MEDLINE;

3. handsearches of 30 journals and the proceedings of major conferences;

4. weekly current awareness alerts for a further 44 journals plus monthly BioMed Central email alerts.

We will also search CENTRAL, MEDLINE and EMBASE to identify trials published since the search cutoff date of 31 August 2008 for the Cochrane review; using the terms [antenatal or prenatal] and [magnesium] and [preterm or premature or neuroprotection or 'cerebral palsy'].

In addition we will access Current Controlled Trials http://www.controlled-trials.com and the Australian and New Zealand Trials Register http://www.anzctr.org.au to identify recently completed or ongoing trials.

Experts in the field and trialists will be asked if they know of any unpublished or other trials.

### Data collection and management

A new set of pre-specified and clearly defined variables (both for participant-level and trial-level factors as well as for outcomes) and a newly developed coding system will be used. Data will be collected on all women randomised coded for anonymity, (date of birth, centre identification); baseline data for descriptive purposes and analyses (reason at risk of preterm birth, gestational age at trial entry, plurality of the pregnancy, expected date of delivery); details of the intervention given (date of randomisation, allocated intervention, type and dose of magnesium sulphate given, mode of administration, whether maintenance dose given, whether re-treatment given and amount); and maternal and infant outcomes listed below to allow planned analyses.

Trialists will provide de-identified IPD in any convenient format by encrypted, electronic transfer where possible or other means as needed. The individual trial data will be recoded as required and stored in a custom designed secure database which will be only accessible by authorised personnel of the AMICABLE Data Management Group. Trialists will be asked to verify all recoded data prior to any analysis and the data will not be used for any other purpose without permission of all collaborators.

Data will be checked with respect to range, internal consistency, missing or extreme values, errors and consistency with published reports. Trial details such as randomisation methods and intervention details will be cross-checked against published reports, trial protocols and data collection sheets. Inconsistencies or missing data will be discussed with the individual trialists and attempts will be made to resolve any problems by consensus. Each trial will be analysed individually, and the resulting analyses and trial data will be sent to the trialists for verification.

We will assess risk of bias for each study using the criteria outlined in the *Cochrane Handbook for Systematic Reviews of Interventions*[47]. These criteria are:

1. Random sequence generation (checking for possible selection bias)

2. Allocation concealment (checking for possible selection bias)

3. i. Blinding of participants and personnel (checking for possible performance bias)

ii. Blinding of outcome assessment (checking for possible detection bias)

4. Incomplete outcome data (checking for possible attrition bias due to the amount, nature and handling of incomplete outcome data)

5. Selective reporting (checking for reporting bias)

6. Other bias (checking for bias due to problems not covered by 1 to 5 above)

We will make explicit judgements about whether studies are at high, low or unclear risk of bias, according to the criteria given in the Handbook; and we will assess the likely magnitude and direction of the bias and whether we consider it is likely to impact on the findings.

### Data items to be collected

#### Trial level information

1. Dates the trial opened and closed accrual

2. Number of women randomised

3. Informed consent procedures

4. Methods of random allocation

5. Stratification factors used

6. Methods of allocation concealment

7. Blinding of outcome assessment

8. Purpose magnesium sulphate treatment given (neuroprotection for the fetus, neuroprotection for the mother, tocolysis, pre-eclampsia, other)

9. Details of the planned intervention in the experimental arm

10. Details of the planned intervention in the control arm

#### Participant-level information: maternal characteristics at trial entry

1. Unique identification coded for anonymity

2. Maternal age

3. Parity

4. Ethnicity

5. Public or private patient

6. Previous obstetric history

7. Reason the woman was considered to be at risk of preterm birth (such as preterm labour/pre-eclampsia/placenta abruption/placenta previa/chorioamnionitis/other antepartum haemorrhage/preterm rupture of membranes)

8. Number of babies in-utero (singleton, twin or higher order multiple pregnancy)

9. Gestational age at trial entry

#### Participant-level information: maternal outcomes after trial entry

1. Actual treatment dose details (loading dose given, maintenance dose, retreatment dose)

2. Time from antenatal magnesium sulphate treatment to birth

3. Adverse events for the woman at time of treatment

4. Intrapartum information

5. Postnatal information

#### Participant-level information: infant outcomes

1. Unique baby identification and mother identification coded for anonymity

2. Date and time of birth

3. Gestational age at birth

4. Gender

5. Mode of birth

6. Birth weight, length, head circumference

7. Apgar score at 1 and 5 minutes

8. Neonatal complications/status

9. Mortality and age at death

10. Cause of death

11. Childhood follow-up assessments

### Planned analyses

A detailed statistical analysis plan will be prepared by the AMICABLE Data Management Group and agreed upon by the AMICABLE IPD Project Team and the AMICABLE Group prior to the data analyses. Any analyses conducted will be based on the checked and updated IPD from all available trials. All randomised participants with outcome data available will be included in the analyses, which will be performed on an intention to treat basis, according to the treatment allocation at randomisation.

For each of the outcomes a one stage approach to analysis will be taken so that the IPD from all eligible trials are included in a single model. Fitting a single model for each outcome variable will enable the variation across trials to be accounted for within the model. A treatment by trial interaction term will be tested to assess heterogeneity of treatment effect across trials. If excessive statistical heterogeneity in treatment effect or inconsistency across trials is detected (i.e. if the trial by treatment interaction term is significant), then the rationale for combining trials will be questioned and the source of heterogeneity explored.

Binary outcomes will be analysed using log binomial regression models and results will be presented as RR with 95% CI and associated two-sided p values. Continuous outcomes will be analysed using linear regression models and results will be presented as differences in means with 95% CI and two-sided p values. Correlation between outcomes due to multiple births will be taken into account using generalized estimating equations as appropriate.

Any differences in treatment effect between prespecified subgroups will be assessed by testing a treatment by subgroup interaction term within the model.

Where data are missing, those patients will be removed from the analysis. Reasons for missingness will be explored where possible. For each outcome if there are unbalanced or large amounts of missing endpoint data for at least one trial then sensitivity analyses will be undertaken to assess the impact of removing such trials from the analysis.

## Outcomes

Outcomes have been chosen to be most representative of the clinically important measures of effectiveness and safety, including serious outcomes, for women and their babies.

### 1. Primary Outcomes

For the infants/children

•death or cerebral palsy

For the women

•any severe maternal outcome potentially related to treatment (death, respiratory arrest or cardiac arrest)

### 2. Secondary Outcomes

For the infants

•gestational age at birth

•birth weight (raw values and Z scores) [48]

•head circumference at birth (raw values and Z scores) [48]

•length at birth (raw values and Z scores) [48]

•Apgar score less than 7 at five minutes

•use of active resuscitation at birth

•use of ongoing respiratory support

•chronic lung disease/bronchopulmonary dysplasia (as defined by trialists)

•neonatal convulsions

•neonatal encephalopathy

•any IVH

•severe IVH (grade 3 or 4)

•cystic periventricular leukomalacia

•posthaemorrhagic hydrocephaly or ventriculomegaly

•proven systemic neonatal infection

•necrotising enterocolitis

•patent ductus arteriosus requiring treatment

•retinopathy of prematurity

For the children (follow up)

•death (fetal, neonatal or later death up to the time of follow up) and cause of death

•cerebral palsy (any cerebral palsy, as defined by the trialists)

•severity of cerebral palsy (categorised as, mild, moderate or severe, as defined by the trialists)

•severe adverse neonatal outcome (defined as death, chronic lung disease, patent ductus arteriosus requiring treatment, neonatal encephalopathy, necrotising enterocolitis, stage 3 or worse retinopathy of prematurity, grade 3 or 4 IVH)

•other neurosensory impairments (other than cerebral palsy)

○developmental delay or intellectual impairment (categorised as nil, mild, moderate or severe, by the trialists)

○blindness (defined as visual acuity worse than 6/60 (20/200) in the better eye)

○deafness (defined as hearing loss requiring amplification or worse)

○gross motor dysfunction (defined as mild, moderate or severe, by trialists or by the Gross Motor Classification System [score 1-5], if available) [49]

○psychomotor dysfunction (categorised as nil, mild (less than 85), moderate (less than 70) or severe (less than 55) by the Psychomotor Developmental Index (PDI)) on the Bayley Scales of Infant Development [50]

•any neurosensory disability (defined as *developmental delay or intellectual impairment *[developmental quotient or intelligence quotient more than one SD below the mean], *cerebral palsy *[abnormality of muscle tone with motor dysfunction], *blindness*, or *deafness*, at follow up later in childhood)

•major neurosensory disability (defined as any moderate or severe neurosensory impairment)

•death or substantial gross motor dysfunction (defined as death, or motor dysfunction (such that the child was not walking at age two years or later, or the inability to grasp and release a small block with both hands at two years or later))

•death or neurosensory disability (defined as death or any of the neurosensory impairments)

•death or major neurosensory disability (defined as death or any moderate or severe neurosensory impairment)

•growth assessments at childhood follow up for:

○weight (raw values and Z scores) [51]

○head circumference (raw values and Z scores) [51]

○height (raw values and Z scores) [51]

•other developmental assessments (as used by the trialists)

For the women

•adverse effects severe enough to stop treatment

•postpartum haemorrhage

•mode of birth

•chorioamnionitis during labour

•intrapartum fever requiring the use of antibiotics

•length of postnatal stay

•individual components of maternal primary outcome

### 3. Planned subgroup analyses

Where data exist, subgroup comparisons will be conducted for the following outcomes;

•death or cerebral palsy

•death

•cerebral palsy

•severe maternal adverse event potentially related to treatment

•death or major neurosensory disability

Any differences in treatment effect between subgroups will be assessed by testing a treatment by subgroup interaction term within the model.

Subgroups

1) primary reason pregnancy was considered to be at high risk of preterm delivery (preterm labour/pre-eclampsia/placenta abruption/placenta previa/chorioamnionitis/other antepartum haemorrhage/prelabour rupture of the membranes)

2) purpose of treatment - neuroprotection of the fetus/neuroprotection of the mother/pre-eclampsia/eclampsia/tocolysis/other)

3) multiple birth

4) gestational age when antenatal magnesium sulphate treatment was given

a) categories: < 26; 26 to < 28; 28 to < 30; 30 to < 32; 32 to < 34 completed weeks at randomisation; (groups will be combined if data are insufficient);

b) categories: < 34; ≥ 34 completed weeks at randomisation;

c) categories: < 32; ≥ 32 completed weeks at randomisation;

d) categories: < 30; ≥ 30 completed weeks at randomisation;

e) categories: < 28; ≥ 28 completed weeks at randomisation.

5) time from antenatal magnesium sulphate treatment to birth (0 to < 4 hours; 4 to < 8 hours; 8 to < 12 hours; 12 to < 24 hours (groups will be combined if data are insufficient)

6) mode of administration (intravenous, intramuscular)

7) actual dose of antenatal magnesium sulphate received

a) actual total trial dose received (< 4 g; 4 to < 14 g; 14 to < 28 g; ≥ 28 g);

b) actual total trial dose received adjusted for pre-trial dose received (< 4 g; 4 to < 6 g; 6 to < 14 g; 14 to < 28 g; ≥ 28 g);

c) pre-trial dose received or not

8) whether maintenance treatment with antenatal magnesium sulphate was used

9) whether repeat antenatal magnesium sulphate treatment was used

### 4. Planned sensitivity analyses

To assess whether the results are robust to trial design, sensitivity analyses will be performed by excluding trials with high rates of participant exclusions, where losses are considered to have the potential to impact on the results.

To assess whether the results are robust to the inclusion or exclusion of trials where IPD are unavailable sensitivity analyses will be undertaken where IPD will be combined with available aggregate data from such trials.

### 5. Multiple comparisons

A large number of outcomes are being investigated in this study which increases the chance of observing 'false positive' results. However, all outcomes are important in giving a full clinical picture that considers the benefits and risks to both mothers and infants. We do not plan formal statistical adjustment of p values planned to account for multiple comparisons due to the non-independence of outcomes in this study. Results will be interpreted with caution.

## Ethics and management issues

### Ethical considerations

Participants in the individual trials have previously given informed consent to participate in their respective trial. The data for this project are to be used for the purpose for which they were originally collected and are available through an agreement between all trialists of the collaborative group. These trialists remain custodians of their original individual trial data at all times.

### Project management

For the purpose of this project, an international Collaborative Group, The AMICABLE Group, was formed and consists of the following working groups with specific responsibilities and tasks:

1. The AMICABLE IPD Project Team

The AMICABLE IPD Project Team is the Steering Group which is responsible for the project's management decisions and the daily management of the Collaboration. The Project Team's tasks are to design the project's protocol and analysis plan, organise The AMICABLE Group Meetings and act as a liaison between all the members of The AMICABLE Group. The AMICABLE IPD Project Team will meet regularly every two to four months, usually by teleconference.

2. The AMICABLE Trialist Group

The AMICABLE Group will consist normally of two members from each eligible trial. The first author for each trial, will be invited to become a member of the Collaboration. In order to keep The AMICABLE Group updated, authors of new trials or previously unknown trials may be contacted and invited to join the Collaboration in the course of the project.

3. AMICABLE Data Management Group

The data management group will be convened by the Chair of The AMICABLE IPD Project Team and comprises the statisticians from the data management centre and participating trials who will conduct the analyses. The data management group will be responsible for the storage and analyses of the IPD project data.

#### AMICABLE Group Meetings

The AMICABLE Group face-to-face meetings will be organised at least twice during the study. Representatives of all eligible trials will be invited to attend the meetings. The meetings will be scheduled, if possible, in conjunction with international conferences. During those meetings, various aspects of the project will be discussed with all the collaborators, such the project's design and conduct, the analysis plan, and the interpretation and reporting of the results. The final collaborators' meeting is scheduled for late 2012.

### Publication of results

The final results of the study will be presented to the collaborators for discussion. The main manuscript will be prepared by The AMICABLE IPD Project Team and circulated to The AMICABLE Group and The AMICABLE Data Management Group for comment and revision. The revised draft paper will be circulated for final comment and agreement prior to publication. Publications will be authored in the name of The AMICABLE Group. The names of all participating collaborators will be acknowledged within the manuscript.

## Discussion

Babies born preterm compared with those born at term have a higher chance of dying in the first few weeks of life. Babies who survive have a high risk of substantial disability from neurosensory impairments such as cerebral palsy, blindness, deafness, or cognitive dysfunction.

The Cochrane review on antenatal magnesium sulphate given to women at risk of preterm birth has identified an important reduction in the risk of cerebral palsy. However it remains unclear what the role of antenatal magnesium sulphate is in clinical practice.

This IPD meta-analysis, using data already collected in the individual trials may reveal which women and/or babies might benefit more from antenatal magnesium sulphate. This approach has been described as the 'gold standard' of systematic review methodology as it allows for more powerful and flexible analysis of both subgroups and outcomes. The international collaboration of trialists will guarantee exceptional data availability and quality, and ensure endorsement and global implementation of recommendations into clinical practice and policy.

The AMICABLE Group was formed in 2009 to undertake a meta-analysis based on IPD. Provision of data by the participating collaborators commenced in 2010 and results will be ready for presentation in 2012.

## Abbreviations

AMICABLE: Antenatal magnesium individual participant data (IPD) international collaboration: assessing the benefits for babies using the best level of evidence; CENTRAL: Cochrane central register of controlled trials; CI: Confidence interval; IVH: Intraventricular haemorrhage; IPD: Individual participant data; RR: Risk ratio.

## Competing interests

The authors declare that they have no competing interests.

## Authors' contributions

The Chair of The AMICABLE Project Team (CAC) wrote the first draft of the IPD protocol and prepared the initial draft of the manuscript. The AMICABLE Project Team and The AMICABLE Data Management Group participated in the protocol development and commented on all drafts of the manuscript. The AMICABLE Group participated in the development of the IPD protocol and have read and approved the final draft of the manuscript.
